# Expert consensus on drug treatment of chronic subdural hematoma

**DOI:** 10.1186/s41016-021-00263-z

**Published:** 2021-11-22

**Authors:** Jianning Zhang, Chinese Society of Neurosurgery, Chinese Medical Association, Chinese Neurosurgical Critical Care Specialist Council, Collaborational Group of Chinese Neurosurgical Translational and Evidence-based Medicine, Zhou Fei, Hua Feng, Guodong Gao, Jiehe Hao, Lijun Hou, Jin Hu, Ying Huang, Baohua Jiao, Hongming Ji, Xiaochun Jiang, Dezhi Kang, Jianrong Li, Xingang Li, Jinfang Liu, Ning Liu, Xianzhi Liu, Ying Mao, Yan Qu, Wai Sang Poon, Ning Su, Tao Sun, Xiaochuan Sun, Jianjun Wang, Renzhi Wang, Junji Wei, Shuo Wang, Gangfeng Yin, Chao You, Rutong Yu, Xinguang Yu, Xianrui Yuan, Jianmin Zhang, Junyi Zhang, Shiguang Zhao, Yuanli Zhao, Zongmao Zhao, Chunlong Zhong, Xide Zhu, Xingen Zhu, Rongcai Jiang, Dong Wang, Ye Tian, Huijie Wei, Wei Quan, Chuang Gao, Shuyuan Yue, Ping Lei, Quanjun Deng, Shu Zhang, Yuan Zhou, Jian Sun, Shuo An, Yingsheng Wei, Xintong Ge

**Affiliations:** 1 Department of Neurosurgery, Tianjin Medical University General Hospital, Tianjin, 300052 China; 2 Key Laboratory of Post-trauma Neuro-repair and Regeneration in Central Nervous System, Ministry of Education, Tianjin, 300052 China; 3 Tianjin Key Laboratory of Injuries, Variations and Regeneration of Nervous System, Tianjin, 300052 China; 4 Tianjin Neurological Institute, Tianjin, 300052 China

## Abstract

Chronic subdural hematoma (CSDH) is a chronic space-occupying lesion formed by blood accumulation between arachnoid and dura mater, which is usually formed in the third week after traumatic brain injury. Surgical treatment is usually the first choice for patients with CSDH having a significant space-occupying effect. Most of the patients showed good results of surgical treatment, but still some patients had a postoperative recurrence (the recurrence rate was up to 33%). Because CSDH is often seen in the elderly, patients are weak and have many basic diseases. The risk of surgical treatment is high; serious complications and even death (the death rate is up to 32%) can often occur. The overall good prognosis rate of patients aged more than 90 years is 24%. The drug treatment can provide a safe and effective treatment for elderly patients who are weak, intolerable to surgery, or failed in surgery. Low-dose and long-term use of atorvastatin (20mg/d) is suggested for continuous treatment for at least 8 weeks, while low-dose and short-term use of dexamethasone can improve the therapeutic effect of atorvastatin on CSDH. Patients should undergo CT or MRI scanning at least one time within 2 weeks after the start of drug treatment.

Chronic subdural hematoma (CSDH) is a chronic space-occupying lesion formed by blood accumulation between arachnoid and dura mater, which is usually formed in the third week after traumatic brain injury. The incidence of CSDH is 1–13.1/100,000, and has increased with the aging of the population. The annual incidence can reach 127/100,000 in elderly people aged more than 80 years [1, 2]. CSDH is generally considered to be caused by trauma, but about 50% of patients deny the history of trauma [3, 4]. In addition to trauma, common risk factors related to the development, malabsorption, or recurrence of CSDH include the following: (1) long-term use of anticoagulants or antiplatelet drugs; (2) repeated, or a sudden increase in, chest and abdominal pressure (such as dystocia and constipation); (3) craniotomy; and (4) hematopathy.

## Pathogenesis of chronic subdural hematoma

The mechanism of CSDH development and absorption is not very clear. A series of studies were carried out on the pathogenesis of CSDH, such as bleeding from the avulsion of the pontine vein, increased osmotic pressure, hematoma capsule hemorrhage, and local hyperfibrinolysis. All studies were considered to be related to the formation and development of CSDH, but the pathogenesis of CSDH is still unclear up to now [5, 6]. Recent evidence shows that trauma and other causes can lead to the accumulation of blood and/or cerebrospinal fluid in the local subdural cavity. Hematoma-derived exosomes promote abnormal angiogenesis with high permeability by delivering miR-144-5p into endothelial cells, which results in re-bleeding and inhibits hematoma absorption [7]. The secretion of inflammatory cytokines and vascular endothelial growth factor (VEGF) leads to the proliferation of immature blood vessels on the hematoma wall, damage to vascular endothelial cells, opening of gap junctions, and increase of permeability. The continuous leakage of circulating substances results in the gradual increase in hematoma growth [6]. At the same time, the lack of anti-inflammatory and pro-repair factors, such as regulatory T (Treg) cells and endothelial progenitor cells (EPCs), leads to the recurrence of “immature angiogenesis–endothelial cell damage–vascular leakage” on the hematoma wall, which may be the key factor for CSDH formation [5, 8–11]. A recent study showed that meningeal lymphatic vessels (mLVs) were also an important pathway for subdural hematoma (SDH) clearance. The presence of SDH hampers the formation and normal functioning of mLVs, which may form a vicious circle and accelerate the accumulation of SDH [12]. These observations indicate that CSDH may be formed because of the malfunction of mLVs and be cleared through mLVs. Other studies have confirmed that immune regulation abnormity and decrease in vascular repair and maturation ability play important roles in the formation and development of CSDH [13–15].

## Clinical manifestations of chronic subdural hematoma

CSDH is a kind of intracerebral hemorrhage disease, which is insidious in onset and slow in progression. The expansion of hematoma leads to intracranial hypertension, resulting in clinical manifestations, such as headache, dizziness, or limb dysfunction. CT scan and MRI can help make a definite diagnosis. The severity of the disease is often evaluated according to the size of hematoma and the degree of midline shift. However, a hematoma develops slowly, and elderly patients with CSDH often have multiple intracranial volume compensation factors, such as brain atrophy. As a result, these patients with more serious imaging performance tend to have mild symptoms and signs (many patients therefore refuse to operate). In addition, disease progression is slow, and the imaging examination cannot meet the needs of clinical dynamics, leading to repeated and timely evaluation. To accurately and timely evaluate the severity and development trend of patients with CSDH, Markwalder’s team proposed the Markwalder’s grading scale and Glasgow Coma Scale (MGS–GCS scale) based on the comprehensive consideration of clinical symptom severity and consciousness state score of patients, as shown in Table 1 [16].

**Table 1:** Definition of Markwalder’s Grading Scale-Glasgow Coma Scale

Patient’s grade	GCS	Markwalder’s Grading Scale
Grade 0	15	Normal neurological status without symptoms
Grade 1	15	Without neurological deficits, but with symptoms such as headache or unsteady gait
Grade 2	13–14	Focal neurological deficits, such as drowsiness or disorientation, or variable neurological deficits, such as hemiparesis
Grade 3	9–12	With stupor but appropriate responses to noxious stimuli and several focal neurological signs such as hemiplegia
Grade 4	< 9	Coma with absent motor responses to noxious stimuli and decerebrate or decorticate posturing

Only patients with grade 0–2 CSDH were selected for atorvastatin treatment in this study

Recommendation 1: MGS–GCS system is recommended to be used as the clinical evaluation standard for the severity of patients with CSDH (moderate-quality evidence, strong recommendation).

Most CSDH often increases gradually. Patients’ conditions continue to worsen with the increase in space-occupying effect; even brain hernia occurs and endangers life (hematoma in a few patients can be absorbed naturally after observation and symptomatic treatment [17]). Surgical treatment is usually the first choice for patients with CSDH having a significant space-occupying effect. Often drilling or burr-hole drainage is chosen [18]. Besides, small bone flap craniotomy or endoscope-assisted evacuation methods are also used [19, 20]. Most of the patients showed good results of surgical treatment, but still some patients had a postoperative recurrence (the recurrence rate was up to 33% [2]). Even patients had to accept multiple surgical treatments or embolization of the middle meningeal artery because of repeated recurrence [21]. Because CSDH is often seen in the elderly, patients are weak and have many basic diseases, leading the long-term mortality rate is high. It belies the notion that CSDH is a benign disease [22]. The overall good prognosis rate of patients aged more than 90 years is 24% [23].

## Drug treatment for chronic subdural hematoma

The aim of drug treatment is to improve symptoms and signs of patients and promote hematoma absorption. Drug treatment can be divided into symptomatic treatment and treatment promoting hematoma absorption. The purpose of symptomatic treatment is to improve neurological symptoms and signs of patients and create conditions for surgery or other treatments. The treatment promoting hematoma absorption can not only provide a simple and less painful treatment for patients but also can be used to prevent a postoperative recurrence. It can provide a safe and effective treatment for elderly patients who are weak, are intolerable to surgery, or failed in surgery.

The indications of drug treatment for promoting hematoma absorption are as follows: (1) vital signs stable, and MGS–GCS grade 0–2; (2) image showing that the midline shift is less than 1 cm, with no need for emergency surgical intervention; (3) patients having multiple-organ failure, coagulation dysfunction, and other unsuitable or refused surgeries; and (4) the recurrence being prevented after surgery. The contraindications are as follows: (1) MGS–GCS grade 3–4; (2) image showing that brain is severely compressed and the midline shift is more than 1 cm; (3) signs of brain hernia, such as consciousness disorder, nausea, and vomiting; and (4) allergy to the used drug or contraindications of the used drug. The recommended drugs for CSDH treatment in this consensus are atorvastatin and dexamethasone. The related contraindications of these two drugs can refer to the product description and are not described in detail. It is suggested that surgical treatment should be used for patients whose clinical manifestations and neuroimages remain unimproved or deteriorate after a 2-week conservative treatment or more; the hematoma continues to increase, or such patients cannot tolerate drugs.

## Atorvastatin therapy for chronic subdural hematoma

Based on the hypothesis of immune regulation disorder and immature angiogenesis, inhibiting an excessive inflammatory response and promoting neovascularization have become a treatment strategy to promote CSDH absorption. The basic studies on traumatic brain injury (TBI) suggest that the increase in EPC mobilization in the circulating blood after TBI can promote the repair of blood–brain barrier, establishment of vascular-nerve unit, and intracranial hematoma absorption, thus improving the prognosis [24]. The clinical observations of patients with TBI confirm that the prognosis of patients with high EPC in the circulating blood is significantly better than that of patients with low EPC [25]. Atorvastatin has been proved to have double regulatory effects of immunoregulation and vascular maturation, which can improve neurological symptoms of rats with TBI [24, 26]. Statins, also termed as 3-hydroxy-3-methylglutaryl coenzyme A (HMG-CoA) reductase selective inhibitors, have previously been used for treating hyperlipidemia. However, statins can also improve the level of circulating EPC and the number of Treg cells, inhibit the nonspecific immune inflammatory response, promote the repair of damaged blood vessels, and enhance the endothelialization of artificial blood vessels (stents). They have been used in the clinical treatment of hypertension and coronary heart disease. The basic studies also found that atorvastatin could inhibit the inflammatory reaction on the wall of CSDH, promote the maturation and repair of immature blood vessels (smooth muscle formation of the vascular wall and stabilization of the gap connection of the endothelial barrier), and accelerate hematoma absorption. However, a high dose of atorvastatin can not only mobilize more EPC but also significantly increase the expression levels of vascular endothelial growth factor (VEGF), tissue growth factor-β (TGF-β), and matrix metalloproteinase-9 (MMP-9). The expression of these factors on the wall of hematoma result in new juvenile vascular hyperplasia and reduce the therapeutic effect of atorvastatin [14, 27]. In clinical practice, the complications of atorvastatin, such as the increase in the levels of liver enzymes and rhabdomyolysis, are found to be closely related to the dosage [28]. Therefore, low-dose and long-term treatment of atorvastatin is more suitable for patients with CSDH.

At present, low-dose atorvastatin has been used by many Chinese neurosurgeons to promote CSDH absorption and prevent the recurrence of CSDH [29–31]. Twenty-five Chinese neurosurgery centers have completed a randomized double-blind controlled trial (RCT) on the use of low-dose and long-term atorvastatin (20 mg/d) for treating CSDH. The results showed that the hematoma volume reduction in the atorvastatin treatment group was 12.55 mL more than that in the control group after 8-week treatment. Most patients’ neurological symptoms significantly improved after drug treatment. At the same time, the transfer to surgery rate decreased significantly in the atorvastatin treatment group. No serious side effects were noted in the course of drug treatment [32]. In addition, this treatment scheme has also been applied to treat some refractory CSDH cases, such as young children who cannot tolerate reoperation due to repeated recurrence and patients with coagulation dysfunction (long-term oral administration of warfarin, aspirin, clopidogrel, and other antithrombotic drugs), and to prevent the postoperative recurrence, achieving a good curative effect [30, 31, 33].

Recommendation 2: For patients with CSDH who meet the indications of drug treatment, low-dose and long-term use of atorvastatin (20 mg/day) is suggested for continuous treatment for at least 8 weeks, until neurological symptoms and signs disappear and hematoma absorption is satisfactory; the drug is then discontinued (high-quality evidence, strong recommendation). It is also suitable for perioperative patients to reduce the recurrence rate (moderate-quality evidence, strong recommendation). The dose of atorvastatin can be increased properly when the blood lipid of patients is still elevated during the treatment period. However, for the sake of safety, it is not more than 80 mg/day (low-quality evidence, strong recommendation).

## Dexamethasone treatment of chronic subdural hematoma

Dexamethasone (DXM) is a synthetic corticosteroid (hormone), which can inhibit the aggregation of immune inflammatory cells, phagocytosis, and release of inflammatory mediators; nonspecifically inhibit the immune inflammatory response; and reduce and prevent the response of tissues to inflammation. According to the needs of patients in neurosurgery (including patients with CSDH), glucocorticoids such as dexamethasone can be used to replace or assist symptomatic treatment. Because dexamethasone has a long half-life and a wide range of dosage, it is widely used clinically [34]. Previous studies confirmed that the indexes of immune inflammation, such as leukocytes in the circulating blood of patients with CSDH, are not high. However, many immune inflammatory cells, inflammatory factors, and other inflammatory reaction products are present in the hematoma wall and cavity of CSDH [35]. In the 1960s, some scholars applied high doses of dexamethasone to treat CSDH [36]. Since then, clinical reports are available on the application of high-dose dexamethasone to promote CSDH absorption and prevent a postoperative recurrence. However, these reports have different treatment courses and dexamethasone doses [37, 38], and they have not been confirmed by reliable, evidence-based medical researches.

Previous reports on the treatment of CSDH with high-dose dexamethasone showed that the dose of dexamethasone was generally 12–16 mg/day, and the total dose of treatment course was more than 336 mg [38]. A high dose of dexamethasone can easily lead to obesity, gastrointestinal damage, and other steroid-related complications. Therefore, patients with hypertension, diabetes, thromboembolism, gastric and duodenal ulcer, psychosis, electrolyte metabolism abnormality, myocardial infarction, glaucoma, and Cushing’s syndrome are generally not suitable for use. Elderly patients, especially women after menopause, are prone to osteoporosis; also, the prevalence of CSDH is high in the elderly. A retrospective study pointed out that high-dose dexamethasone (6–8 mg/day) could only save 17% of patients with CSDH from surgery, but significantly increased treatment complications [39]. Recent evidence-based medical research (meta-analysis) showed that the dosage of dexamethasone used in CSDH treatment was large, and its side effects were worrying; hence, the rate of its use declined [40].

Atorvastatin can inhibit the local inflammatory response of CSDH and promote vascular repair, achieving the therapeutic purpose. It can reduce the vascular leakage caused by the CSDH hematoma fluid, but its effect is weak. In clinic, low-dose and short-term dexamethasone treatment refers to the daily dose of 0.5–3 mg/day, and the duration is no more than 4 weeks (the total dose of dexamethasone is about 60 mg) [33]. Low-dose and short-term use of dexamethasone with atorvastatin can enhance the inhibition of vascular leakage caused by an inflammatory reaction and avoid adverse effects of long-term use of dexamethasone. Therefore, low-dose and short-term use of dexamethasone with atorvastatin is expected to better correct the imbalance between damage factors and repair factors in CSDH. One proof of concept (POC) study confirmed that the effect of low-dose atorvastatin combined with low-dose and short-term dexamethasone on CSDH was more significant compared with the single use of low-dose atorvastatin, and it did not increase drug-related side effects. Although a rigorous RCT study has yet to be designed to further confirm the actual efficacy of this combined therapy, this POC showed that the combined therapy could be used as a priority therapy for patients with CSDH without contraindications of drug use, providing a new choice for patients with poor efficacy of atorvastatin monotherapy [41].

Recommendation 3: High-dose (12–16 mg/day) or long-term (more than 3 months) routine use of dexamethasone is not recommended due to its large side effects (high-quality evidence, strong recommendation); low-dose and short-term use of dexamethasone can improve the therapeutic effect of atorvastatin on CSDH. For patients with a refractory or repeated recurrence of CSDH and without any obvious curative effect of single-use atorvastatin, it is recommended to combine dexamethasone (the first dose is 2.25 mg/day, lasting for 1–2 weeks, gradually reduced and discontinued within 4 weeks) and low-dose atorvastatin (20 mg/day) and then continue to receive low-dose atorvastatin (20 mg/day) until neurological symptoms and signs disappear, and hematoma absorption is satisfactory (low-quality evidence, strong recommendation).

## Hemostasis and antifibrinolytic therapy of chronic subdural hematoma

CSDH is an intracranial bleeding disease. Long-term use of oral anticoagulants and antiplatelet drugs are high-risk factors for CSDH [42]. A recent evidence-based medical study (meta-analysis) showed that restarting anticoagulant therapy (excluding antiplatelet drugs) after surgery would increase the risk of CSDH recurrence [43]. For patients who take anticoagulants and antiplatelet drugs for a long time, relevant drugs should be stopped immediately after CSDH is confirmed; it is difficult to stop anticoagulant and antiplatelet drugs due to stent placement, artificial vascular replacement, and heart valve replacement. Blood coagulation function and platelet-related monitoring should be carried out, and antagonists can be used if necessary. After CSDH hematoma is completely absorbed, patients’ previous use of anticoagulant and antiplatelet drugs should be restored and the coagulation function should be closely monitored. For the specific treatment principles, please refer to the relevant consensus [44].

CSDH has a high incidence in the elderly, who often have a variety of systemic thrombotic diseases, such as cerebral infarction and coronary atherosclerotic heart disease. The application of hemostatic drugs greatly increases the risk of thrombotic diseases. Hemostatic drugs are not usually used.

The antifibrinolytic therapy of CSDH has received increasing attention. The studies showed that tissue plasminogen activator, fibrin degradation products, and thrombomodulin in hematoma fluid and outer membrane of patients with CSDH increased significantly, suggesting that hyperfibrinolysis is related to repeated blood leakage from blood vessels [45–48]. Antifibrinolytic drugs can stop bleeding by inhibiting plasminogen activation and plasminogen activity. Clinical case reports and retrospective studies have shown that tranexamic acid, as an antifibrinolytic drug, can be used to treat CSDH, promote hematoma absorption, and reduce recurrence [49–53]. However, some studies believed that antifibrinolytic drugs could increase the incidence of thrombotic events in patients [54]. Therefore, more reliable high-level evidence-based medical research is needed for antifibrinolytic drug treatment.

Recommendation 4: Once patients with CSDH are diagnosed, anticoagulants and antiplatelet drugs should be stopped in principle (very low-quality evidence, strong recommendation). In patients with systemic coagulation disorders, hemostatic drugs should be used carefully (very low-quality evidence, strong recommendation). In addition, before a high level of evidence-based medical research, antifibrinolytic drugs are not recommended (low-quality evidence, weak recommendation).

## Drug treatment for chronic subdural hematoma complication

1. Drug treatment for CSDH combined with intracranial hypertension and headache: When CSDH compresses and stimulates brain tissue, it can lead to a headache, nausea, and other symptoms of high intracranial pressure, as well as movement and language dysfunction, and mental symptoms. The treatment using osmotic dehydrating drugs such as mannitol, glycerin fructose, and diuretics can relieve the pain. However, a well-regulated RCT confirmed that osmotic therapy did not lead to hematoma absorption [55]. For patients with obvious headache symptoms, nonsteroidal anti-inflammatory drugs, such as acetaminophen, naproxen, and ibuprofen, can be used. Before and during the treatment of osmotic therapy, it is necessary to monitor the renal function and the change in electrolytes, so as to prevent the aggravation of renal insufficiency and the electrolyte disorder among elderly patients [56]. The use of opioid drugs should be reduced. At the same time, regular imaging monitoring should be carried out to pay attention to changes in intracranial hematoma and brain edema.

(2) Drug treatment for CSDH combined with depression, anxiety, and mental and behavior disorder: Some patients with CSDH and mental symptoms, such as anxiety, depression, and insomnia, can use flupenthixol melitracen, diazepam, estazolam, tandospirone, and zaleplon to regulate mood and sleep [57]. Olanzapine is often used to control mental and emotional symptoms, and the dosage of statins should be adjusted for patients with elevated blood lipid levels during the treatment period [58]. However, the application of the aforementioned drugs may cause serious side effects, and patients need to be informed of the risk when the drugs are used for a long time.

(3) Drug treatment for CSDH combined with epilepsy: Epilepsy is one of the main complications before and after CSDH drilling and drainage. The incidence of epilepsy can reach 3–23% after CSDH is diagnosed. The clinical treatment of epilepsy usually involves the use of sodium valproate and other antiepileptic drugs. However, the effect of prophylactic use of antiepileptic drugs is uncertain. The prophylactic use of antiepileptic drugs for patients with CSDH is still controversial. No randomized controlled and recent retrospective studies have evaluated the risk and benefit of the prophylactic use of antiepileptic treatment in patients with CSDH. However, for the elderly and alcoholic patients, the prophylactic use of antiepileptic treatment may be safer and more beneficial [59].

Recommendation 5: Osmotic therapy is recommended to treat the increase in intracranial pressure caused by CSDH (moderate-quality evidence, strong recommendation), but its use is not recommended to promote CSDH absorption (high-quality evidence, strong recommendation). Antidepressants and sleep aid drugs are recommended to improve the mood and insomnia symptoms among patients with CSDH (moderate-quality evidence, strong recommendation). Olanzapine is recommended to treat patients with mental and emotional disorders (low-quality evidence, strong recommendation). Sodium valproate is recommended to be used as a therapeutic drug for epilepsy in patients with CSDH (moderate-quality evidence, strong recommendation), but it is not recommended to prevent epilepsy in nonepileptic patients (low-quality evidence, weak recommendation).

## Evaluation and monitoring of the effectiveness of drug treatment for CSDH

Patients with CSDH who are ineffective in drug treatment often show the aggravation or continuous unremitting of the original neurological symptoms and signs; the imaging shows that hematoma gradually increases. These patients may develop brain herniation due to the increase in hematoma, and therefore, they need to be transferred to surgery in time. The effective treatment for CSDH is shown to be the alleviation of original neurological symptoms and signs, and/or the decrease in hematoma on neuroimaging. For some elderly patients, the volume of hematoma was reduced to a certain extent and no longer reduced. However, the neurological symptoms and signs completely disappeared and the long-term follow-up did not change, which could be regarded as effective treatment.

In addition, about 15% of patients taking atorvastatin have elevated transaminase levels, and a small number of patients have abnormal alkaline phosphatase and bilirubin levels; very few patients have rhabdomyolysis symptoms [60]. However, some patients may have increased heart rate, facial flush, appetite, pore size, and body mass after low-dose dexamethasone treatment. It may also aggravate the condition of patients with diabetes, peptic ulcer, osteonecrosis of the femoral head, and osteoporosis. Therefore, all patients receiving CSDH drug treatment need to receive strict monitoring of clinical neurological symptoms and signs, neuroimaging, and blood test.

Recommendation 6: It is recommended that all patients with CSDH receiving drug treatment should be accompanied for 24 h. In the case of aggravation of neurological symptoms, they should go to the nearest neurosurgery emergency department for medical treatment (low-quality evidence, strong recommendation). Patients should undergo CT or MRI scanning at least one time within 2 weeks after the start of drug treatment (low-quality evidence, strong recommendation). Patients should undergo blood routine examination, liver and kidney function tests, and blood lipid and blood glucose tests within 2 weeks after the start of drug treatment, and creatine kinase and myoglobin tests if necessary (low-quality evidence, strong recommendation).

**Fig. 1:**
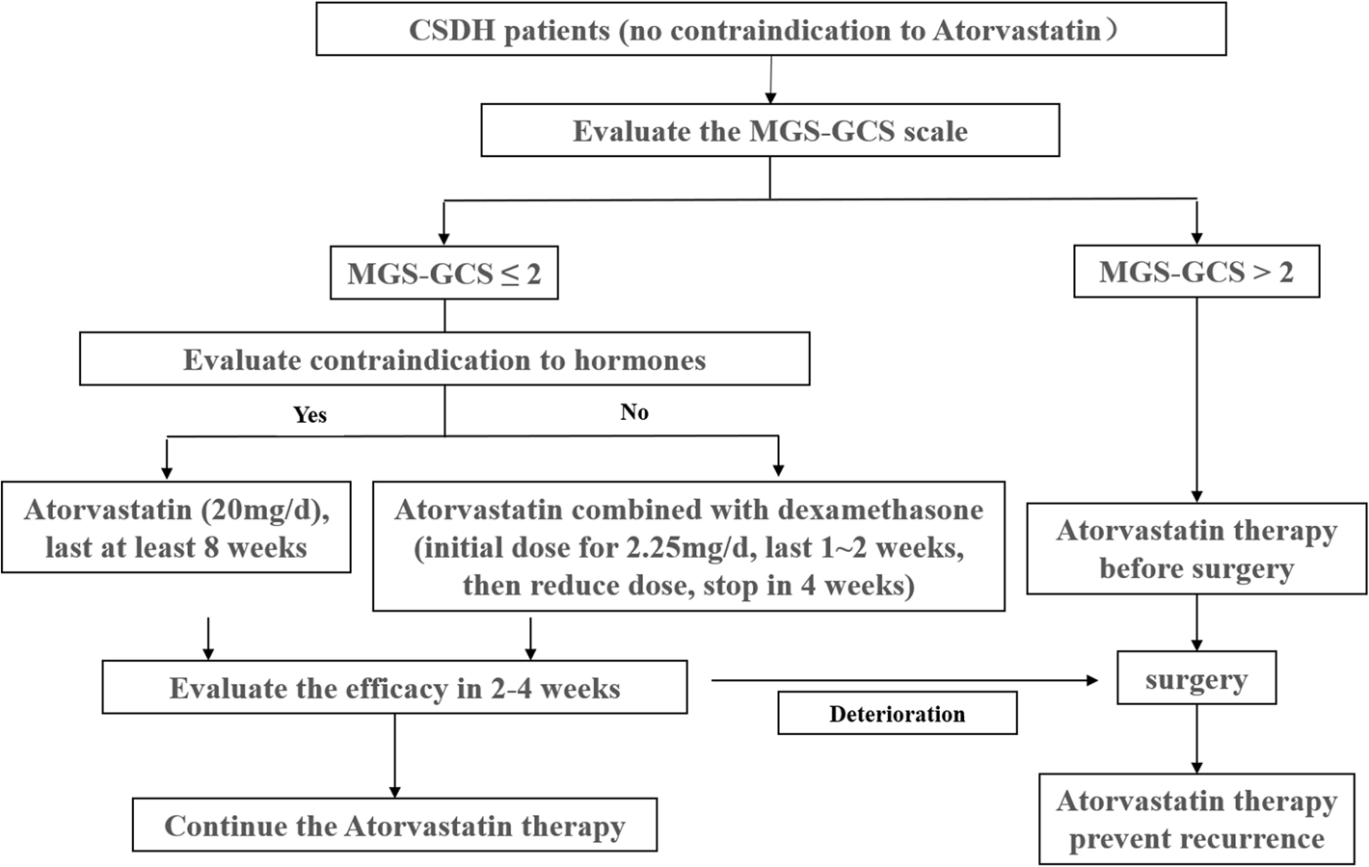
Flow chart of CSDH drug treatment

According to the above consensus, the flow chart of CSDH drug treatment is shown in Fig. 1.

## Explanation

1. Up to now, the exploration of drug therapy to promote CSDH absorption has gone through a long developmental process. In addition to atorvastatin and dexamethasone, researchers have tried to use a variety of drugs or even herbal drugs, but these drug therapies either have uncertain efficacy and huge adverse reactions, or have not been tested in clinical RCTs; safe and reliable evidence is lacking [61]. Therefore, this consensus does not recommend these drugs.
2. In this consensus, the clinical and basic evidence regarding the use of atorvastatin in the treatment of CSDH is obtained based on the application of Lipitor. The clinical evidence is mainly from adult samples. It is not confirmed that other brands of atorvastatin and other types of statins also have equivalence. It cannot be confirmed that the long-term oral administration of atorvastatin can reduce the incidence of CSDH, and can still achieve good results.
3. This consensus recommendation is applicable to adult patients with CSDH. For children, please refer to the literature [33].
4. The understanding of the pathogenesis and treatment of CSDH has gradually deepened with the increasing attention of neuroscience clinical and scientific researchers to this disease and the extensive development of multidisciplinary collaborative research. New effective treatment methods, including new drugs and new surgical methods, may be used in the future. This consensus will also be updated with time, looking forward to a greater breakthrough in the research on CSDH.
5. This consensus is based only on the literature available at present and the evidence-based medical evidence held by experts participating in the discussion. It is only for the reference of neurosurgery clinicians, does not have a legal effect, and does not serve as the legal basis for any medical disputes and lawsuits. The right of interpretation lies in the committee of experts on the preparation of this consensus.
6. In this consensus, the evidence quality is divided into four levels: high, moderate, low, and very low, and the recommendation level is divided into two levels: strong recommendation and weak recommendation. See Table 2 for the GRADE classification method [62].

**Table 2:** GRADE classification method

Quality level	Current definition
High	We are very confident that the true effect lies close to that of the estimate of the effect
Moderate	We are moderately confident in the effect estimate: The true effect is likely to be close to the estimate of the effect, but there is a possibility that it is substantially different
Low	Our confidence in the effect estimate is limited: The true effect may be substantially different from the estimate of the effect
Very low	We have very little confidence in the effect estimate: The true effect is likely to be substantially different from the estimate of effect
Recommendation level	
Strong	Benefits clearly outweigh risk, or vice versa
Weak	Uncertainty in the estimates of benefits and risks; benefits and risk may be closely balanced

## Conclusions

CSDH is recommended to evaluate the severity of patients by MGS-GCS system. Low-dose and long-term use of atorvastatin (20mg/d) is suggested for continuous treatment for at least 8 weeks, while low-dose and short-term use of dexamethasone can improve the therapeutic effect of atorvastatin on CSDH. Patients should undergo CT or MRI scanning at least one time within 2 weeks after the start of drug treatment.

## Data Availability

Not applicable
